# A New Model for Brain Tumor Detection Using Ensemble Transfer Learning and Quantum Variational Classifier

**DOI:** 10.1155/2022/3236305

**Published:** 2022-04-14

**Authors:** Javeria Amin, Muhammad Almas Anjum, Muhammad Sharif, Saima Jabeen, Seifedine Kadry, Pablo Moreno Ger

**Affiliations:** ^1^Department of Computer Science, University of Wah, Wah 47040, Pakistan; ^2^National University of Technology (NUTECH), Islamabad, Pakistan; ^3^Department of Computer Science, Comsats University Islamabad, Wah Campus, Wah 47040, Pakistan; ^4^Department of IT and Computer Science, Pak-Austria Fachhochschule: Institute of Applied Sciences and Technology, Haripur, Pakistan; ^5^Department of Applied Data Science, Noroff University College, Kristiansand, Norway; ^6^Professor, Vice-Rector for Research, Universidad Internacional de La Rioja, Logroño 26006, La Rioja, Spain

## Abstract

A brain tumor is an abnormal enlargement of cells if not properly diagnosed. Early detection of a brain tumor is critical for clinical practice and survival rates. Brain tumors arise in a variety of shapes, sizes, and features, with variable treatment options. Manual detection of tumors is difficult, time-consuming, and error-prone. Therefore, a significant requirement for computerized diagnostics systems for accurate brain tumor detection is present. In this research, deep features are extracted from the inceptionv3 model, in which score vector is acquired from softmax and supplied to the quantum variational classifier (QVR) for discrimination between glioma, meningioma, no tumor, and pituitary tumor. The classified tumor images have been passed to the proposed Seg-network where the actual infected region is segmented to analyze the tumor severity level. The outcomes of the reported research have been evaluated on three benchmark datasets such as Kaggle, 2020-BRATS, and local collected images. The model achieved greater than 90% detection scores to prove the proposed model's effectiveness.

## 1. Introduction

In both adults and children, brain tumors are one of the major causes of mortality [1]. American Brain Tumour Association (ABTA) states that about 612,000 westerners are diagnosed with a brain tumor [2–4]. The term tumor, also known as a neoplasm, refers to the irregular tissue expansion that occurs when cells grow abnormally [5]. Two major types of brain tumors have been identified, each of which is based on the location of the tumors (primary and metastatic) as well as their malignancy growth types (benign and malignant) [6]. Magnetic resonance imaging (MRI) has an ability to discern the tiniest features inside the body. When managing brain lesions and other tumors, MRI is commonly used. We may predict anatomical details and locate anomalies using MRI. This method is better than computed tomography at detecting changes in tissue or structures, and it can also detect a size of a tumor [7]. Manual brain tumor detection is a tough task. As a result, using computer vision techniques to automate brain tumor segmentation and categorization is critical [8]. The clinical approaches allow for the extraction of relevant data and a thorough study of images, whereas computational approaches provide aid in deciphering the nuances in medical imaging [9]. The precise morphology assessment of tumors is a vital task for better treatment [10–12]. Despite substantial work in this sector, physicians still rely on manual tumor determination leading to a shortage of communication among researchers and doctors [13]. Many strategies for automatic classification have been presented lately, which may be divided into feature learning and evaluation processes [14–18]. Recent deep learning techniques, particularly CNN, have shown to be accurate and are frequently employed in medical picture analysis. Furthermore, they have disadvantages over traditional approaches in that they require a big dataset for training, have a high time complexity, are less efficient in application with a limited dataset, and require significant GPUs, all of which raise user costs [19, 20]. Selecting the correct deep learning tools is particularly difficult because it necessitates an understanding of many parameters, training methods, and topology [21].

### 1.1. Motivation

Although a great deal of study has gone into detecting brain tumors, there are still limits in this area due to the complicated pattern of the lesion's locations. The detection of the small amount of lesions region is a great challenge because the small region also appears as a healthy region. Furthermore, extracting and selecting informative features is a difficult task because it directly minimized the classification accuracy. Convolutional neural networks provide help for informative features extraction, but these models are computationally exhaustive. Still, there is a need for a lightweight model for the analysis of brain tumors [22–24].

Therefore, to overcome the existing limitations, this research presents a unique method for more accurately segmenting and classifying brain lesions. The following is the salient contributory steps of the proposed model:

- At the very first contributing step, the score vector is created from the pretrained inceptionv3 model and passed to a six-layer quantum model that trained on tuned parameters such as the number of epochs, batch size, and optimizer solver for prediction among the different classes of the brain tumor such as glioma, no tumor, pituitary tumor, and meningioma.
- An improved Seg-Network has been developed and trained on selected tuned parameters with actual segmented ground masks. It segments the tumor region more precisely.

The following is a summary of the overall article structure: related work is examined in Section 2, while proposed methodology steps are defined in Section 3. The results and discussion are further elaborated in Section 4, and the conclusion is given in Section 5.

## 2. Related Work

A lot of research is done to detect brain tumors, and some of the most current findings are covered in this section. A fuzzy rough set with statistical features is utilized for medical image analysis [25]. Possibilistic fuzzy-c-mean is used with texture features for breast anomalous detection [26–28]. Contrast enhancement is applied to improve the image contrast, and identification of the edges is done using fuzzy logic with dual complex wavelet transform. Furthermore, classification of meningioma/nonmeningioma is performed using U-network [29]. The fine-tuned ResNet-18 model is used for deep feature extraction on 138 subjects of Alzheimer's, and it provides 99.9% classification accuracy [30]. Image fusion plays a vital role in the diagnostic process. MR and CT slices are fused with a sparse convolutional decomposition model. In this, contrast stretching and gradient spatial method are used for edge identification; furthermore, texture cartoon decomposition is employed to create a dictionary where improved sparse convolutional coding with decision maps is used to obtain a final fused slice. Outcomes are evaluated on six benchmark datasets that reflect better performance [31]. A capsule neural model has been designed for brain tumor classification at 86.56% correct prediction rate [32]. The pretrained models such as inceptionv3, ResNet-50, and VGG-16 have been utilized for tumor classification, in which competitive outcomes are achieved on the ResNet-50 model [33]. A hybrid model has been developed in which VGG-net, ResNet, and LSTM models are merged for tumor cell classification that provides 71% accuracy rate on Alex and ResNet and 84% on the VGG16-LSTM model [34]. The singular decomposition value is employed for tumor classification and provides 90% sensitivity (SE), 98% specificity, and 96.66% accuracy [35]. A new model has been proposed based on DWT for tumor classification and has achieved 93.94% accuracy [36]. The histogram equalization approach has been employed to enhance the image quality. Informative features are selected by PCA and passed to the feed-forward network for normal/abnormal MRI image classification with a 90% accuracy rate [37]. The SVM model has been employed for tumor classification that achieved 82% sensitivity and 81.48% specificity. The combination of DWT, PCA, and SVM has been applied for tumor classification that achieved an 80% correct prediction rate with 84% SE and 92% specificity rate [38]. Three machine learning (ML) models have been employed for tumor classification. The model achieved an accuracy of the 88% [39]. A modified CNN model is used for classification of tumors using capsule network (CapsNet). The suggested CapsNet takes advantage of the tumor's spatial interaction including its neighbouring tissues [40]. Another study used the DBN to distinguish between healthy patients and controls with schizophrenia, using 83 and 143 patients from the Radiopaedia database, respectively [40]. In comparison to SVM, which delivers 68.1% accuracy, the suggested DBN gives 73.6% accuracy [41]. A method has been suggested for classifying all types of brain tumors [42, 43]. Likewise, a new framework has been created for classifying brain cancers. To extract the features, the suggested model includes six layers [44, 45]. A multiclass CNN model has been developed for tumor classification. An adversarial generative model has been utilized for the creation of synthetic images [46]. There are six layers in the suggested paradigm. This was combined with a variety of data enhancement techniques. On specified and random splits, this attained an accuracy of 93.01% and 95.6%, respectively [47]. Several additional architectures have lately been proposed to generalize a CNN, particularly in the classification of medical images [48].

Different authors [49, 50] have chosen the graph CNN (GCNN) for tumor classification. Despite different proposed approaches for classifying brain tumors, these methodologies have a number of drawbacks, which have been mentioned in [51, 52]. Many techniques for classifying tumors relied on manually specified tumor regions, preventing them from being entirely automated [53]. The algorithms that used CNN and its derivatives were unable to deliver a significant speed boost. As a result, performance evaluations based on indicators other than precision become increasingly important. Furthermore, CNN models perform poorly on tiny data sets [54–56]. To overcome the existing limitations in this work, a lightweight quantum neural model is proposed for brain tumor classification.

## 3. Proposed Methodology

**Figure 1:**
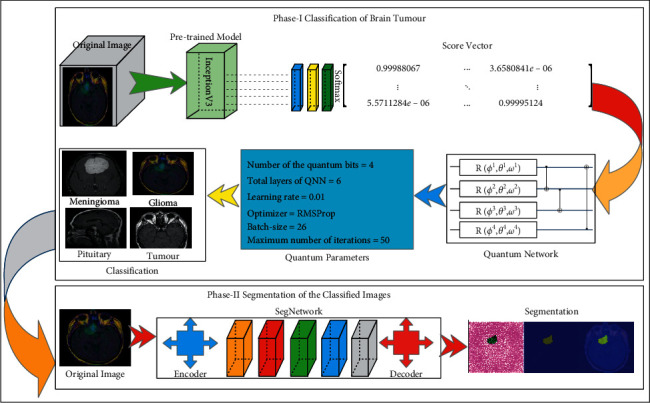
The proposed model for brain tumor detection.

The suggested strategy is divided into three stages such as feature extraction, classification, and segmentation. In phase-I, features are extracted using the pretrained inceptionv3 model. The obtained score vector is further passed to the quantum learning mechanism for tumor classification. In the segmentation phase, SegNetwork has been utilized to segment the actual tumor lesions. The proposed semantic diagram is shown in Figure 1.

Figure 1 shows the processes of the proposed model, which include extracting features using a pretrained inceptionv3 model and obtaining a score vector from the softmax layer. The six-layer quantum network is trained with the score vector based on selected hyperparameters for tumor classification in phase-I. Furthermore, in phase-II, tumor slices are segmented using the proposed semantic segmentation model.

### 3.1. Features Extraction of the Pretrained Model

The features are taken from the inceptionv3 model that consists of 315 layers, in which 94 convolutional, 94 ReLU, 94 batch-normalization, 04 max-pooling, 09 average pooling, 01 global pooling, fully connected, output classification, 15 depth concatenation, and softmax. Based on the probability, classification has been done using softmax. In this layer, *ϕ*_input_*i*__ denotes the *i*_th_ class probability. The class prediction comprises the maximum probability *p*, in which N denotes the total number of classes as follows:

(1) $$\begin{array}{c} \varphi_{\mathrm{input}}=\frac{p^{x_{i}\mathrm{inpu}t_{i}}}{\sum_{J=1}^{N}p^{x_{i}\mathrm{inpu}t_{i}}}. \end{array}$$

The score vectors have been obtained from the softmax and supplied as input to the quantum model for classification.

#### 3.1.1. Classification of Brain Tumours Based on the Score Using the Quantum Model

Demand for processing power growth is another issue where computationally costly applications include simulations of massive quantum systems such as molecules and solving massive linear problems. This is a major reason for the creation of quantum computing, a computational approach that uses the properties and concepts of quantum particles to handle data. Quantum computers offer an exponential speedup. Although quantum computers have advanced quickly in recent years, theoretical and practical difficulties still stand in the way of a large-scale computing device. Related to noisy processes, quantum computers currently have severe constraints, such as restricted qubits and operations of gates. Variational quantum algorithms (VQAs) [57] have emerged as one of the most promising approaches to overcome these constraints. This technique has been presented in a variety of disciplines, including object recognition as a quantum machine learning algorithm. This study presented a brain tumor classification methodology based on the VQA-based data reuploading classifier (DRC).

Quantum computers, like conventional computers, utilize operation gates to regulate and modify the configuration of a qubit. The unitary matrix may be used to explain the quantum gates mathematically. Unitary evolution is the term used to describe the transition from one quantum state to another via gates of quantum. The physics of the qubit is implementation, reliant on the hardware design (hardware) of such gates of the quantum. Each implementation of a qubit has its unique method for generating gates of quantum. Two or even more qubits might be used to run quantum gates. A quantum circuit, or an array of several quantum gates working on greater than one qubit, can be used to run a quantum method. Qubits is a state of qubit after working on a gate of the quantum. Assume that the Hadamard gate (H) operation on the qubit |0 outputs in a qubit inside the following state of superposition:

(2) $$\begin{array}{c} \mathrm{Hadamard}\;\mathrm{gate}|0\rangle =\frac{1}{\sqrt{2}}\left[\begin{array}{cc} 1 & 1\\ 1 & -1 \end{array}\right]\left[\begin{array}{c} 1\\ 0 \end{array}\right]=\frac{1}{\sqrt{2}}\left[\begin{array}{c} 1\\ 1 \end{array}\right]=\frac{1}{\sqrt{2}}\left(\left[\begin{array}{c} 1\\ 0 \end{array}\right]+\left[\begin{array}{c} 0\\ 1 \end{array}\right]\right)=\frac{1}{\sqrt{2}}|0\rangle +\frac{1}{\sqrt{2}}|1\rangle ,\\ \text{CNOT}\left(\left(\text{Hadamard gate}|\text{0}\rangle \right)⊗|\text{0}\rangle \right)=\frac{1}{\sqrt{2}}|\text{0}\rangle +\frac{1}{\sqrt{2}}|11\rangle . \end{array}$$

The control is shown by black dots on the schematic depiction of the CNOT gateway, while the target is represented by the “×” symbol within the circle. If the controlled qubit is in the |1〉 state, the gateway of CNOT will invert the quantum state of qubit target to |0〉 to |1〉 and vice versa. Parametric gates of quantum operate based on parameters that are placed on gates. RX, RY, and RZ gates are parametric having the following matrix functional:

(3) $$\begin{array}{c} RX\left(\phi \right)=e^{-\frac{i\phi \sigma_{x}}{2}}=\left[\begin{array}{cc} \cos\left(\phi /2\right) & -i\;\sin\left(\phi /2\right)\\ -i\;\sin\left(\phi /2\right) & \cos\left(\phi /2\right) \end{array}\right], \end{array}$$

where *e* represents the epochs,

(4) $$\begin{array}{c} RY\left(\phi \right)=e^{-\frac{i\phi \sigma_{y}}{2}}=\left[\begin{array}{cc} \cos\left(\phi /2\right) & -i\;\sin\left(\phi /2\right)\\ -i\;\sin\left(\phi /2\right) & \cos\left(\phi /2\right) \end{array}\right],\\ RZ\left(\phi \right)=e^{-\frac{i\phi \sigma_{z}}{2}}=\left[\begin{array}{cc} e-\left(\phi /2\right) & 0\\ 0 & e\left(\phi /2\right) \end{array}\right]. \end{array}$$

The (*ϕ*), (*ϕ*), and (*ϕ*) gates rotate the qubit vector around the x-rotation axis, *y*-rotation axis, and *z*-rotation axis as in the Bloch sphere image. The qubit vector is then rotated on three axes of rotation with varying angle values using three parametric gates. Consider the rotation gate (*ϕ*), which has given a matrix structure whose function is dependent on the following parameters:

(5) $$\begin{array}{c} R\left(\phi ,\theta ,w\right)=\left[\begin{array}{cc} e^{-i\left(\phi +w\right)/2}\;\cos\left(\theta /2\right) & -e^{i\left(\phi -w\right)/2}\;\sin\left(\theta /2\right)\\ e^{-i\left(\phi -w\right)/2}\;\sin\left(\theta /2\right) & e^{-i\left(\phi +w\right)/2}\;\cos\left(\theta /2\right) \end{array}\right]. \end{array}$$

Here, *w* denotes weights.

**Figure 2:**
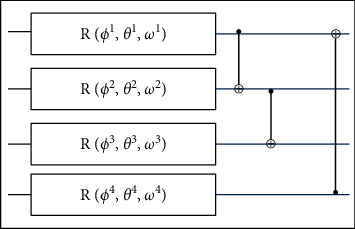
Structure of four-qubit method.

This gate spins a qubit around the *z*-axis, then the *y*-axis, and finally back to the *z*-axis in the Bloch representation. The softmax layer of the inceptionv3 model generates the score vector, which is then given to the variational quantum model. In the proposed model, 4-qubit structure is utilized for model training. The schematic diagram of the four-qubit structure is illustrated in Figure 2.

This method employs four qubits, including one with an operation of gate *R*, preceded by a succession of CNOT gates. The total parameters of this ansatz version are 12 since each gate *R* has three parameters. The parameters are tuned, like ANN, such that the circuit can undergo unitary evolution, resulting in the specific intention findings. The model is trained on the parameters stated in Table 1.

Table 1 shows the learning QNN parameters in which four quantum bits, six QNN layers, 0.01 learning rate with the RMSProp optimizer solver, and a batch size of 26 are utilized on 50 training epochs.

### 3.2. Proposed Seg-Network

In this study, the improved Seg-Network model is provided that consists of 59 layers, in which 16 convolutional, 16 ReLU, 16 batch-normalization, 4 maxpooling, and a softmax. SegNet is made up of two subnetworks: an encoder and a decoder. The number of times that the input layer is downsampled or upsampled as it is processed is determined by the depth of the networks. The encoder part of the network downsamples the resolution of the input image through the 2*D* factor, where *D* denotes the encoder depth, and the output of the decoder network is upsampled by a factor. For each segment of the SegNet encoder model, the output channels are given as just a positive integer/vector. SegNet layers adjust the decoder's output channel count to match the encoder portion. The biased factor is set to zero in convolutional layers. The segmentation model's learning parameters are provided in Table 2.

## 4. Results and Discussion

The proposed method was tested on three distinct types of benchmark datasets in this study, including The Cancer Genome Atlas (TCGA), BRATS 2020, and locally gathered photos.

TCGA data contain 101 cases: precontrast and postcontrast and Flair. In these data, nine cases of the postcontrast sequence are missing and six cases are missing from the sequence of precontrast [58, 59]. The binary masks of this dataset are in a single channel. The suggested technique is also tested on benchmark datasets like privately gathered photographs from a local hospital. The input MRI ground masks are also manually generated by professional radiologists, in which 800 tumors/nontumor slices of 20 patients are included with the dimension of 320 × 320 × 600, where 320 × 320 represents the image dimension and 600 denotes the number of slices across each patient.

The 2020-BRATS Challenge incorporates data from 259 patients of MRI, with 76 cases of low glioma grades and 76 high glioma grades [60–62]. Every patient has 155 MRI slices with a dimension of 240 × 240 × 155.

The multiclass brain tumor classification dataset has been downloaded from the Kaggle website. This dataset contains four classes such as glioma, meningioma, no tumor, and pituitary tumor. The dataset contains two folders, one for training and another one for testing. The glioma, meningioma, no tumor, and pituitary tumor contain 826, 822, 395, and 827 training slices, respectively. However, testing slices of glioma, meningioma, no tumor, and pituitary tumor are 100, 115, and 74, respectively [63].

The proposed study has been evaluated on MATLAB toolbox with 2021 RA with a Windows 10 operating system. The proposed method's performance was evaluated based on two experimentations. The classification model's performance was evaluated in the first experiment. The second experiment was used to assess the effectiveness of the segmentation approach.

### 4.1. Experiment#1: Classification of Brain Tumours

**Figure 3:**
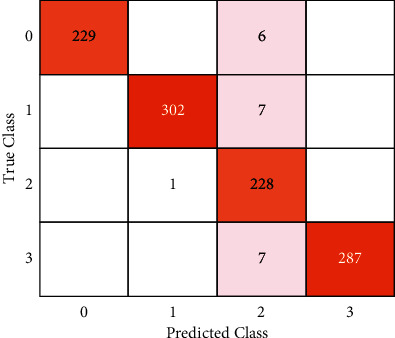
Confusion matrix.

In this experiment, classification data are split into two halves, in a 70 : 30 ratio. The computed classification results on benchmark datasets are given in Table 3. The classification outcomes are also plotted in the form of confusion matrices as shown in Figure 3.

**Figure 4:**
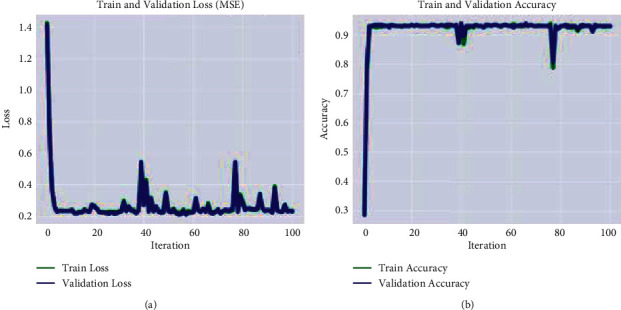
Graphical representation of the model training on the Kaggle dataset. (a) MSE. (b) accuracy.

Figure 3 shows the ratio of true positive and true negative values, where 0, 1, 2, and 3 shows the no tumor, meningioma, pituitary tumor, and glioma, respectively. The training and validation loss rate with respect to the accuracy is plotted in Figure 4.

**Figure 5:**
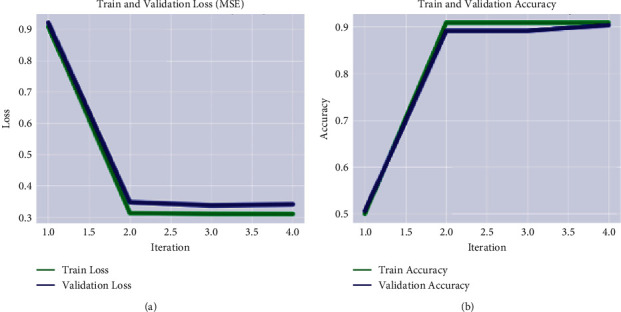
Graphical representation of model training on the Kaggle dataset. (a) MSE. (b) accuracy.

Table 3 shows the calculated recognition accuracy, which demonstrates that the obtained multiclass accuracy is 99.44% on no tumor, 99.25% on meningioma, 98.03% on the pituitary, and 99.34% on glioma. The classification of low- and high-grade glioma results is also computed on BRATS-2020 as depicted in Table 4. The training and validation loss rate with respect to the accuracy is depicted in Figure 5.

**Figure 6:**
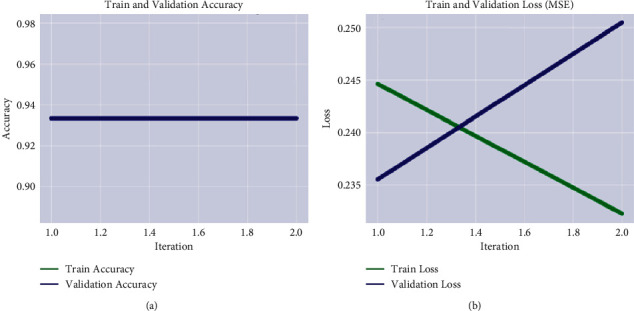
Graphical representation of model training on local collected images. (a) MSE. (b) accuracy.

Table 4 shows the classification outcomes on BRATS-2020 Challenge where the proposed method achieved 90.91% accuracy. The classification results on local collected images from POF Hospital are as shown in Table 5. The graphical representation of training and validation progress is plotted in Figure 6.

Table 5 depicts the classification outcomes that is 93.33% accurate on local collected images. The outcomes of the proposed classification are compared to current works as stated in Table 6.

Binary grading classifier is used for classification of HGG/LGG on BRATS-2020 dataset and provides an accuracy of 84.1% [64]. Features are extracted from the RNN model for the classification of tumor grades such as pituitary tumor [65] and meningioma and give an accuracy of 98.8% [66]. A deep network is developed for brain tumor classification on the Kaggle dataset, and it provides an accuracy of 97.87% [67]. The CNN model is trained on the tuned hyperparameters using the Kaggle dataset, and it gives an accuracy of 96.0% [68]. GRU, LSTM, RNN, and CNN models are used as base learner with a min-max fuzzy classifier for classification, and this provides an accuracy of 95.24% [69].

The proposed approach based on convolutional and quantum neural network provides an accuracy of 98.2% on the Kaggle dataset and 99.7% on the BRATS-2020 Challenge dataset.

In the future, this work might be enhanced for the volumetry analysis of brain tumors that will help the radiologists in the diagnostic process.

### 4.2. Experiment#2: Segmentation of the Brain Tumor

**Figure 7:**
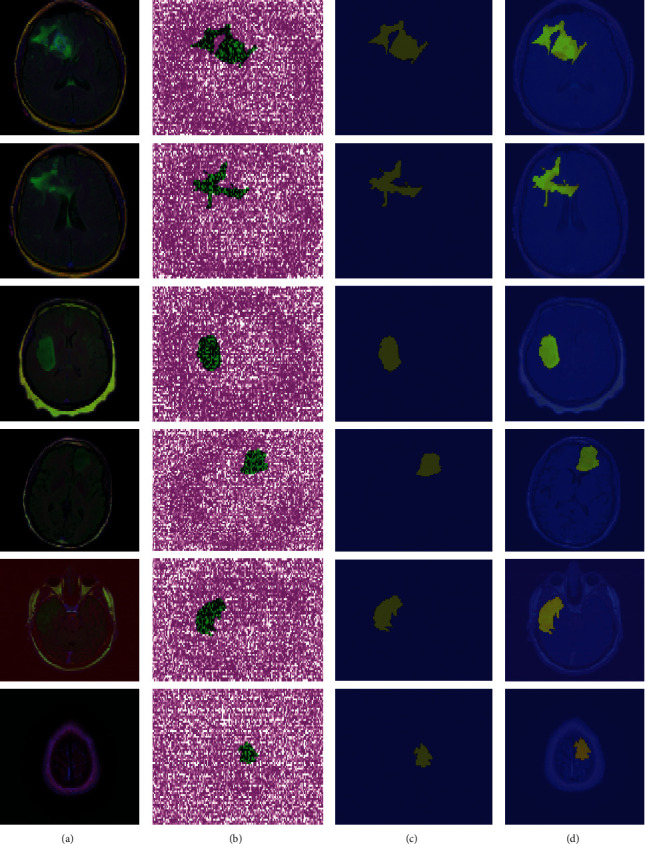
Proposed method segmentation outcomes on the Kaggle dataset (a). Original MRI slices (b). Three-dimensional segmentation (c). The segmented region is mapped on input slices (d).

**Figure 8:**
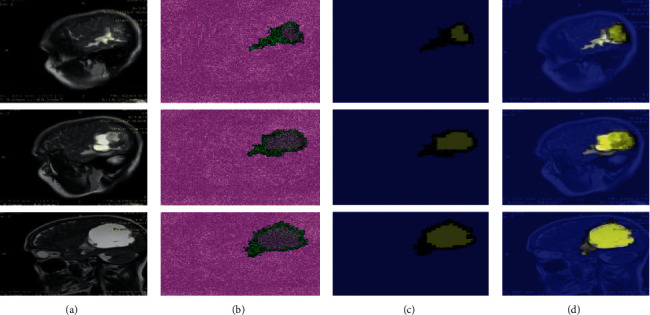
Proposed method segmentation outcomes on local collected images (a). Original MRI slices (b). Three-dimensional segmentation (c). The segmented region is mapped on input slices (d).

**Figure 9:**
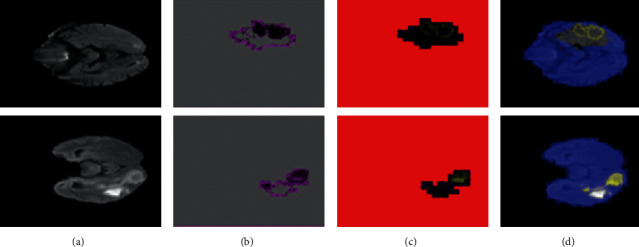
Proposed method segmentation outcomes on Challenge BRATS-2020 (a). Original MRI slices (b). Three-dimensional segmentation (c). The segmented region is mapped on input slices (d).

In this experiment, Table 7 shows the results of testing the suggested segmentation algorithm on benchmark datasets. Figures 7–9 illustrate the results of the proposed segmentation approach.

Table 7 depicts the segmentation outcomes, where the proposed approach achieved a global accuracy of 0.982 on the Kaggle dataset, 0.997 on Challenge BRATS-2020, and 0.999 on images collected from local hospitals. As indicated in Table 8, the obtained segmentation results are compared to the most recent techniques.

Simulation results of the proposed method have been compared with those of eight latest existing works (as seen in Table 8). Noise is eliminated from the MRI slices with an anisotropic filter, and we classify the tumor/healthy slices using SVM, which provides 96.04% prediction scores [70]. A nonlocal mean filter is used for noise reduction, and tumor pixels are segmented by region growth. The classification of tumor/normal slices is done by a neural network classifier on the Kaggle dataset with 97.3% prediction scores [78]. Morphological operations with a Gaussian filter are applied on MRI slices to segment the brain tumor on the Kaggle dataset, and it gives 93% prediction scores [72]. A fine-tuned transfer learning network is employed for tumor classification on Kaggle data, and it provides 92.67% prediction scores [73]. Deep features are extracted from pretrained Resnet50, in which the five last layers are replaced with the eight new layers, and they give 97.2% prediction scores [74]. The suggested ME-Net classification model consists of the multiple encoder and decoder layers for classification of the tumor slices, and it provides prediction scores of 70.20% [75]. A generic three-dimensional network is designed for the classification of MRI slices, and it provides 88.90 prediction scores on the BRATS-2020 dataset [76]. The skull is removed from MRI slices by region-based approaches, and adaptive FTE clustering is used to segment the tumor pixels. The robust discrete wavelet, HOG, and intensity features are extracted from each MRI slice, and we classify the healthy/unhealthy images using DENN which provides prediction scores of 97% [79].

The experimental analysis clearly shows that the proposed approach performed far better. This work will be enhanced in the future for the classification of tumor substructure such as whole tumor, enhanced tumor structure, and nonenhanced tumor structure to analyze the tumor severity rate.

## 5. Conclusion

Because of the complicated nature of the lesion's area, detecting malignancy grades is a difficult process. In this research study, an improved idea is provided for classifying and segmenting brain tumors at an initial stage, to enhance patient survival rate. In this work, features are retrieved using the inceptionv3 model and obtained score vector by softmax that is further passed to the variational quantum classifier for brain tumor classifications. The performance of the classification method is evaluated on two publicly available datasets and one local dataset. On the Kaggle dataset, it achieved an accuracy of 99.44% on no tumor, 99.25% on meningioma, 98.03% on pituitary tumor, and 99.34% on glioma. In the classification between tumor and nontumor classes, the proposed method achieved 93.33% accuracy on local collected images. On the 2020-BRATS Challenge, the proposed method attained an accuracy of 90.91% with HGG and LGG slices. After classification for analyzing the total infected region of the tumor, a modified Seg-Network has been introduced and it provides a global accuracy of 0.982 on the Kaggle dataset, 0.999 on private collected images, and 0.997 on the 2020-BRATS Challenge. The experimental study shows that the proposed model outperformed the most recent published work in this field. In the future, this research will improve the capacity and capability of the health sector in general, as well as local hospitals, for early diagnosis resulting in a higher survival rate.

## Figures and Tables

**Table 1:** Learning parameters of the QNN model.

Number of the quantum bits	4

Total layers of the quantum neural network (QNN)	6
Learning rate	0.01
Optimizer	RMSProp
Batch size	26
Maximum number of iterations	50

**Table 2:** Initial segmentation learning parameters.

Size of the input	240 × 240⟶ 2020 BRATS
	240 × 240⟶ TCGA
	240 × 240⟶ local data

Optimizer	Sgdm
Number of classes	02
Depth of the encoder	4
Rate of learning	1*e* − 3
Training epochs	100

**Table 3:** Multiclass classification results on the Kaggle dataset.

Classes	Acc.	Precision	Recall	F1 score

No tumor	99.44%	0.97	1.0	0.99
Meningioma	99.25%	0.98	1.0	0.99
Pituitary	98.03%	1.0	0.92	0.96
Glioma	99.34%	0.98	1.0	0.99

**Table 4:** Classification of HGG/LGG results on the 2020-BRATS Challenge dataset.

Classes	Acc.	Precision	Recall	F1 score

HGG	90.91%	0.87	0.94	0.91
LGG	90.91%	0.95	0.88	0.91

**Table 5:** Classification of normal/abnormal results on local collected images.

Classes	Acc.	Precision	Recall	F1 score

Tumor	93.33%	0.93	0.93	0.93
Nontumor	93.33%	0.93	0.93	0.93

**Table 6:** Comparison of the proposed approach to existing methodologies.

Ref#	Year	Dataset	Type of tumors	Achieved outcomes

[64]	2021	BRATS-2020	HGG/LGG	84.1% acc.
Proposed method				90.91% acc.

[66]	2021	Kaggle	Glioma, meningioma, no tumor, and pituitary tumor	98.21% acc.
[67]				97.87% acc.
[68]				96% acc.
[69]	2022			95.24% acc.
Proposed method				99.10% acc.

**Table 7:** Proposed tumour segmentation results.

Data	Global acc.	Mean acc.	Mean IoU	Weighted IoU	Mean BF score

Kaggle	0.98201	0.93235	0.94402	0.97397	0.98358
Local collected	0.99975	0.99975	0.99988	0.99975	0.99783
BRATS-2020	0.99781	0.85378	0.93876	0.99757	0.83913

**Table 8:** Comparison of the simulated improved Seg-network results to the existing methods.

Ref#	Year	Dataset	Achieved outcomes%

[70]	2021	Kaggle	96.04
[71]			97.30
[72]			93.00
[73]	2022		92.67

Proposed method			98.20

[74]	2020	BRATS 2020	97.20
[75]	2021		70.20
[76]	2020		88.90
[77]	2022		99.15
Proposed method			99.70

## Data Availability

The multiclass brain tumor classification dataset has been downloaded from the Kaggle website. This dataset contains four classes such as glioma, meningioma, no tumor, and pituitary tumor (“Navoneel Chakrabarty, Swati Kanchan, https://github.com/sartajbhuvaji/brain-tumor-classification-dataset (accessed on 6 June 2021”). The 2020-BRATS Challenge incorporates data from 259 patients of MRI, with 76 cases of low and high glioma grades. Every patient has 155 MRI slices with a dimension of 240 × 240 × 155 (B. H. Menze, A. Jakab, S. Bauer, J. Kalpathy-Cramer, K. Farahani, J. Kirby, et al., “The multimodal brain tumor image segmentation benchmark (BRATS),” IEEE transactions on medical imaging, vol. 34, pp. 1993–2024, 2014. S. Bakas, H. Akbari, A. Sotiras, M. Bilello, M. Rozycki, J. S. Kirby, et al., “Advancing the cancer genome Atlas glioma MRI collections with expert segmentation labels and radio atomic features,” Scientific data, vol. 4, p. 170117, 2017. M. Kistler, S. Bonaretti, M. Pfahrer, R. Niklaus, and P. Büchler, “The virtual skeleton database: an open access repository for biomedical research and collaboration,” Journal of medical Internet research, vol. 15, *p*. e245, 2013).
